# Protective Role of P2Y_2_ Receptor against Lung Infection Induced by Pneumonia Virus of Mice

**DOI:** 10.1371/journal.pone.0050385

**Published:** 2012-11-21

**Authors:** Gilles Vanderstocken, Els Van de Paar, Bernard Robaye, Larissa di Pietrantonio, Benjamin Bondue, Jean-Marie Boeynaems, Daniel Desmecht, Didier Communi

**Affiliations:** 1 The Institute of Interdisciplinary Research, IRIBHM, Université Libre de Bruxelles, Brussels, Belgium; 2 The Department of Pathology, Faculté de Médecine Vétérinaire, Université de Liège, Liège, Belgium; 3 The Institute of Interdisciplinary Research, IRIBHM, Université Libre de Bruxelles, Gosselies, Belgium; 4 The Department of Pneumology, Erasme Hospital, Université Libre de Bruxelles, Brussels, Belgium; 5 The Department of Laboratory Medicine, Erasme Hospital, Université Libre de Bruxelles, Brussels, Belgium; Louisiana State University Health Sciences Center, United States of America

## Abstract

ATP released in the early inflammatory processes acts as a danger signal by binding to purinergic receptors expressed on immune cells. A major contribution of the P2Y_2_ receptor of ATP/UTP to dendritic cell function and Th2 lymphocyte recruitment during asthmatic airway inflammation was previously reported. We investigated here the involvement of P2Y_2_ receptor in lung inflammation initiated by pneumonia virus of mice infection. We demonstrated that P2Y_2_^−/−^ mice display a severe increase in morbidity and mortality rate in response to the virus. Lower survival of P2Y_2_^−/−^ mice was not significantly correlated with excessive inflammation despite the higher level of neutrophil recruiters in their bronchoalveolar fluids. Interestingly, we observed reduced ATP level and lower numbers of dendritic cells, CD4^+^ T cells and CD8^+^ T cells in P2Y_2_^−/−^ compared to P2Y_2_^+/+^ infected lungs. Lower level of IL-12 and higher level of IL-6 in bronchoalveolar fluid support an inhibition of Th1 response in P2Y_2_^−/−^ infected mice. Quantification of DC recruiter expression revealed comparable IP-10 and MIP-3α levels but a reduced BRAK level in P2Y_2_^−/−^ compared to P2Y_2_^+/+^ bronchoalveolar fluids. The increased morbidity and mortality of P2Y_2_^−/−^ mice could be the consequence of a lower viral clearance leading to a more persistent viral load correlated with the observed higher viral titer. The decreased viral clearance could result from the defective Th1 response to PVM with a lack of DC and T cell infiltration. In conclusion, P2Y_2_ receptor, previously described as a target in cystic fibrosis therapy and as a mediator of Th2 response in asthma, may also regulate Th1 response protecting mice against lung viral infection.

## Introduction

Acute viral bronchiolitis represents a major challenge in both developing and industrialized countries. Indeed, amongst many viruses who can induce bronchiolitis, studies have shown that respiratory syncytial virus is the cause of 70% of all cases of viral bronchiolitis [1]. Human respiratory syncytial virus (hRSV) is a negative-sense, single-strand RNA virus of the family *Paramyxoviridae*. hRSV is the most common cause of airway morbidity among children under 1 year of age and can cause subsequent infections throughout life [2].

Infection of mice with pneumonia virus of mice (PVM) is used as a natural host experimental model for studying the pathogenesis of infection with the closely related hRSV [3]–[5]. PVM infection induces a disease that begins on day 6 post-infection and inoculation of more than 300 PFUs is generally lethal by day 9 post-infection [6]. The primary targets of PVM *in vivo* are respiratory epithelial cells [7]. In infected mice, virus replication is accompanied by a profound inflammatory response with recruitment of granulocytes, marked edema, mucus production, and airway obstruction, leading to significant morbidity and mortality [7]–[10]. This is associated with marked respiratory dysfunction and by local production of inflammatory mediators including MIP-1α (CCL3), MIP-2 (CXCL2), MCP-1 (CCL2) and IFN-γ [7]. Subsequently, a predominant Th1 adaptive response occurs from day 8 post-infection, with a pronounced influx of CD8^+^ cytotoxic T cells [11], [12]. This cytotoxic response is enhanced by type I interferon production (IFN-α and IFN-β) and plays a crucial role in anti-PVM immunity, as it contributes to control PVM replication and is correlated to the severity of the disease in a viral dose-dependent fashion.

Metabotropic P2Y receptors have been recognised as important regulators of cell functions [13]–[15]. Amongst the P2Y receptors family, P2Y_2_ is an ubiquitous receptor that is fully activated by ATP and UTP [16]. Metabotropic receptors are coupled to intracellular signalling pathways through heterotrimeric G proteins [15]. Several studies have demonstrated that extracellular nucleotides regulate lung inflammation: P2Y_1_ and P2Y_2_ receptors exert a protective role against infection of the lungs by *P. aeruginosa* [17] and P2Y_2_ was described as a target for cystic fibrosis therapy [18]. Moreover, the role of ATP in eosinophil recruitment and dendritic cell activation during asthma has been previously shown [19]. We have previously shown that P2Y_2_ receptor acts also as a regulator of membrane and soluble forms of VCAM-1 mediating the adhesion and migration of eosinophils in an asthma model [20].

In this study, we investigated the consequences of P2Y_2_ loss in lung inflammation initiated after PVM infection.

## Materials and Methods

### Ethics statement

This study was carried out using mice in strict accordance with the national, european (EU Directives 86/609/EEC) and international guidelines in use at the Université Libre de Bruxelles. All procedures were reviewed and approved by the ethics committee (Commission d'Ethique du Bien-Etre Animal, CEBEA) of the Université Libre de Bruxelles (Permit Number: 338N, 146N and 341N). All efforts were made to minimize suffering: mice were placed in a ventilated room with all appropriate hygiene and feeding conditions throughout the experiments. Housing, inoculation, data collection, and euthanasia procedures complied with National Institutes of Health guidelines, and the experimental protocol was approved by the Bioethics Committee of the University of Liège. Mice were monitored twice daily and in the case of a weight loss exceeding 30% of the body weight or clear signs of animal suffering, mice were euthanized by cervical dislocation and integrated in the mortality curve data.

### Animals

P2Y_2_R knockout (P2Y_2_^−/−^) mice were provided as breeder pairs (on a B6D2 genetic background) by Dr. B.H. Koller (University of North Carolina, Chapel Hill, NC). The B6D2 P2Y_2_^−/−^ mice were then crossed with the SV129 mouse strain by Dr. J. Leipziger (Institute of Physiology and Biophysics, University of Aarhus, Aarhus, Denmark), generating B6D2/SV129 P2Y_2_^+/+^ and B6D2/SV129 P2Y_2_^−/−^ littermates. Mice were then backcrossed onto C57Bl6 for >10 generations. The experiments were conducted with specific pathogen-free 8-week-old female mice

### PVM inoculation

PVM strain J3666 (generously supplied by A. Easton, University of Warwick, Coventry, UK) was first passed in 10-week-old BALB/c mice and then grown once onto BS-C-1 cells to produce the stock solution. The stock solution was then diluted to 10^−5^ in MEM, divided into aliquots, and stored at−80°C to serve as inoculum. Randomly selected aliquots yielded highly reproducible titers on BS-C-1 cells, amounting to ≈5×10^5^ PFU/mL. The inoculation procedure consisted of slowly instilling 50 µL of the viral suspension into the nostrils of the anesthetized mouse maintained in a vertical position (35 mg/kg pentobarbital sodium intraperitoneally). Mice were inoculated under brief anaesthesia (ketamine, Pfizer, 50 mg/kg, and xylazine, Bayer, 10 mg/kg, i.p.) by intranasal instillation of 50 µl of a viral suspension containing 1000 PFU and 1% BSA in PBS [10].

At selected time intervals (8, 9, 10 and 12 day post-infection), groups of minimum 6 mice were sacrificed with sodium thiopental (5 mg/animal, i.p.) and exsanguination. Broncho-alveolar lavage fluids (BALFs) were obtained by flushing the lungs with sterile 0.9% NaCl, and cell counts were performed on cytospin preparations after Diff-Quick staining (Dade Behring, Deerfield, IL) and by flow cytometry.

### Quantification of ATP level in the BALF of PVM-infected mice

P2Y_2_^+/+^ and P2Y_2_^−/−^ mice were infected with PVM and their BALF was collected at day 8, 9 and 10 post-infection. ATP level was quantified in the BALF using the luminescence ATP detection assay system ATPlite (PerkinElmer, Zaventem, Belgium) as previously described [20].

### Quantification of leukocyte infiltration by flow cytometry analysis

Flow-cytometry data acquisition was performed on a dual-laser FACSCalibur flow cytometer running CELLQuest software (BD Biosciences, Erembodegem, Belgium). WinMDI software was used for data analysis. Cells were stained with mAbs directed against F4/80 (FITC), CD11b (PerCp-Cy 5.5), CD4 (PerCP), CD11c (APC), I-A/I-E (PE), CD19 (PE), Ly-6G and Ly-6C (PerCp-Cy 5.5), CD8 (FITC), CD3 (APC), and isotype controls (all from BD Biosciences except F4/80-FITC from AbD Serotec).

### Quantification of cytokine levels in BALFs of P2Y_2_^+/+^ and P2Y_2_^−/−^ mice

Cytokines such as KC, MIP-2, MIP-1α, MCP-1, IL-12p40, IFN-γ, TNF-α, IL-6, IFN-β, IL-17, MIP-3α, IP-10 were measured in P2Y_2_^+/+^ and P2Y_2_^−/−^ BALFs using ELISA kits from BD Biosciences and R&D Systems (Abingdon, U.K.), following the manufacturer's instructions. BRAK was measured by RT-qPCR using the following primers set: 5′-GAT GAA GCG TTT GGT GCT CT-3′ and 5′-AGT ACC CAC ACT GCG AGG AG-3′, with Power SYBR Green PCR Master Mix (Applied Biosystem). Reactions were run on a 7500 Fast Real-Time PCR System (Applied Biosystems). The cycling conditions were 10 min for polymerase activation at 95°C and 40 cycles at 95°C for 15 s and 60°C for 60 s. Mean ± SD values were obtained for each gene using qBase software. Each assay was performed in duplicate.

### Viral Titration

At day 8 and day 10 post-infection, mice were euthanized to quantify lung virus titers by quantitative polymerase chain reaction (qPCR) as previously described [21]. The lungs were homogenized in ice-cold BSA 1% in PBS, and clarified (1000 g for 10 min). Viral RNA was extracted using Nucleospin RNA Virus columns according to the user manual (Macherey Nagel). Homogenates were treated with Fermentas DNase I and an aliquot of each RNA extract (100 ng RNA) was then reverse-transcribed using commercial high capacity cDNA reverse transcription kit (Invitrogen), and PCR was conducted using the following PVM SH gene primers set: 5′-GCC GTC ATC AAC ACA GTG TGT-3′ and 5′-GCC TGA TGT GGC AGT GCT-3′, with SYBR green PCR Master Mix (Applied Biosystem). SDHA was selected as control gene after analysis for its stability in our system. Reactions were run on a 7500 Fast Real-Time PCR System (Applied Biosystems). The cycling conditions were 10 min for polymerase activation at 95°C and 40 cycles at 95°C for 15 s and 60°C for 60 s. Mean ± SD values were obtained using qBase software. Each assay was performed in duplicate.

### Histological analysis of inflamed lungs of P2Y_2_^+/+^ and P2Y_2_^−/−^ mice

Left lungs were insufflated with 700 µl of 4% paraformaldehyde, and embedded in paraffin. Sections (7 mm) were stained with haematoxylin and eosin and assessed by light microscopy. Histological analysis has been performed according to the score related to PVM infection defined by Anh et al [10]. Lung slides have been examined independently and blindly by two individuals.

### Statistical analysis

For all experiments, data are presented as mean ± S.E.M. and the statistical significance between samples was calculated using the Student's t test or one-way analysis of variance, using the Prism 5 software (GraphPad). The normal distribution of the data was checked using Kolmogorov–Smirnov, D'Agostino–Pearson, and Shapiro–Wilk tests. Kaplan-Meier survival curves were compared using the Log-rank (Mantel-Cox) Test and the Gehan-Breslow-Wilcoxon Test.

## Results

### Survival and weight loss of P2Y_2_^+/+^ and P2Y_2_^−/−^ mice after lung infection with Pneumonia Virus of Mice (PVM)

**Figure 1:**
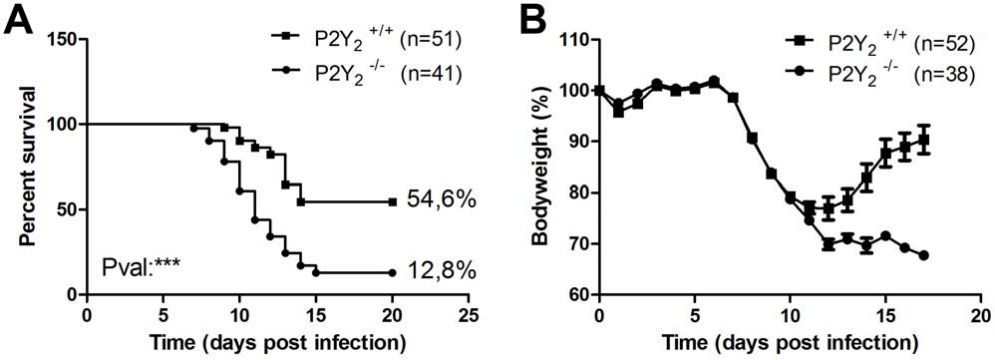
Higher mortality rate in PVM-infected P2Y_2_-deficient mice. Following intranasal inoculation of PVM (1000 PFUs), P2Y_2_^+/+^ and P2Y_2_^−/−^ mice were monitored daily for survival (**A**) and weight loss (**B**). Weight curves (mean ± SEM) are relative to initial body weight. The displayed data result from the pooling of four independent experiments.

To assess the role of P2Y_2_R in the lung, 8-week-old P2Y_2_ wild-type (WT) and P2Y_2_^−/−^ mice were infected with 1000 plaque-forming units (PFUs) of PVM and were monitored daily for survival (Fig. 1A) and weight loss (Fig. 1B). Mice recovered quickly from the anaesthesia and appeared to be normal over the first 6 days. P2Y_2_^+/+^ and P2Y_2_^−/−^ mice began to lose weight on the same day (at day 7, Fig. 1B). Thereafter, the weight curves were roughly the same until day 11, when P2Y_2_^+/+^ mice stabilized their weight before recovering, whereas most of the P2Y_2_^−/−^ mice continued to lose weight 1 day more and then stabilized before death (Fig. 1A and 1B). Survival of P2Y_2_^−/−^ mice was thus significantly decreased compared to P2Y_2_^+/+^ mice (12.8% (N = 51) vs. 54.6% (N = 41) respectively; ***; p<0.001) (Fig. 1A). Furthermore, from day 7 post-infection, infected P2Y_2_^−/−^ mice exhibited a more severe pattern of illness signs, characterized by motor slowing, hunching, and groans.

### Cellular infiltration in the lungs of PVM-infected P2Y_2_^+/+^ and P2Y_2_^−/−^ mice

**Figure 2:**
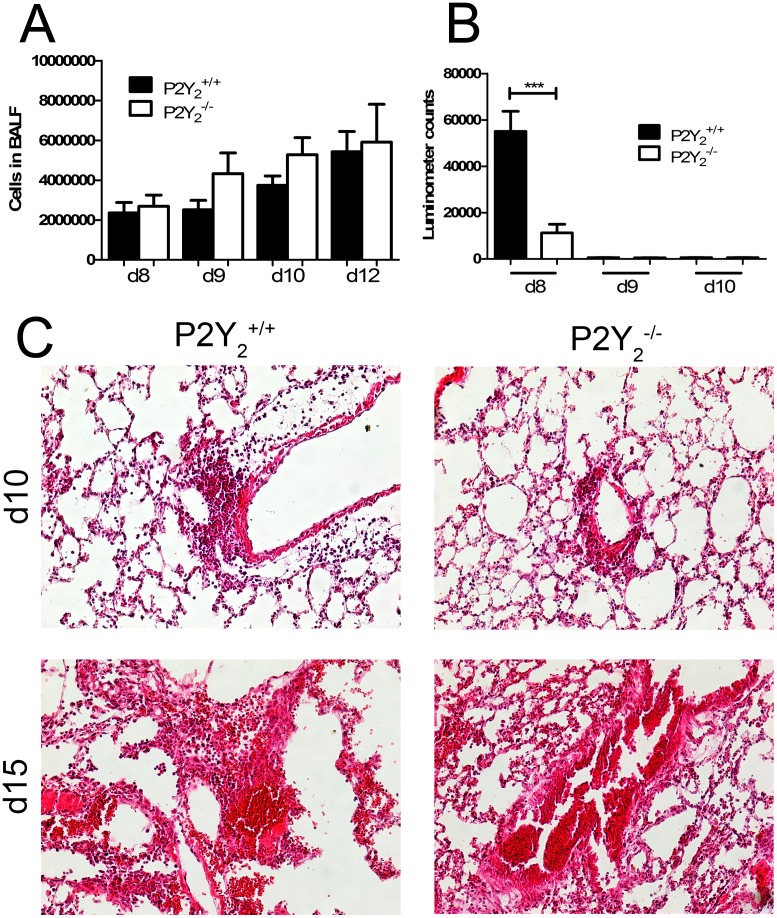
Cellular infiltration in the lungs of PVM-infected P2Y_2_^+/+^ and P2Y_2_^−/−^ mice. 50 µL of the viral suspension were instilled into the nostrils of the anesthetized mouse maintained in a vertical position as described in Materials and Methods. **A**, BALF was collected and the total number of cells was evaluated at days 8, 9, 10 and 12 (N = 7). **B.** Quantification of ATP level in the BALF of PVM-infected P2Y_2_^+/+^ and P2Y_2_^−/−^ mice. ATP level was quantified in the BALF of P2Y_2_^+/+^ and P2Y_2_^−/−^ mice using ATP detection assay system ATPlite at d8, d9 and d10 post-infection with PVM. **C**, Histological analysis of representative lungs of P2Y_2_^+/+^ and P2Y_2_^−/−^ mice 10 days and 15 days after infection with PVM. Paraffin sections (7 µM) of lungs of P2Y_2_^+/+^ and P2Y_2_^−/−^ mice infected by PVM were stained with haematoxylin-eosin (magnification: ×200).

To determine if the difference of survival between P2Y_2_^+/+^ and P2Y_2_^−/−^ mice was due to an excess of inflammation, absolute BALF cell numbers were determined by microscopy and the total cell counting was similar in P2Y_2_^+/+^ and P2Y_2_^−/−^ mice (Fig. 2A). ATP level was then quantified in the BALF of P2Y_2_^+/+^ and P2Y_2_^−/−^ mice using ATP detection assay system ATPlite at day 8, day 9 and day 10 post-infection (Fig. 2B). A lower ATP content was detected in P2Y_2_^−/−^ compared to P2Y_2_^+/+^ infected lungs at day 8 whereas no significant level of ATP was detected at day 9 and day 10 in P2Y_2_^+/+^ and P2Y_2_^−/−^ infected lungs (Fig. 2B).

Microscopic examination of the lungs has been performed at day 10 and day 15 post-infection (Fig. 2C). Histological analysis has been performed according to the score related to PVM infection defined by Anh et al [10]. Lesions were graded from “N” (normal) to “+++”. The value “+” corresponds to scattered inflammatory cells, the value “++” corresponds to dispersed clusters of inflammatory cells and the value “+++” corresponds to dispersed confluent areas of inflammatory cells and severe structure alterations. All P2Y_2_^+/+^ and P2Y_2_^−/−^ infected lungs have been scored “++” at day 10 (N = 5, 6 fields per lung) and “+++” at day 15 (N = 3; 6 fields per lung). The number of P2Y_2_^−/−^ mice surviving until day 15 was very low. Severe loss of lung structure was observed in both P2Y_2_^+/+^ and P2Y_2_^−/−^ mice between day 10 and day 15 post-infection (Fig. 2C).

**Figure 3:**
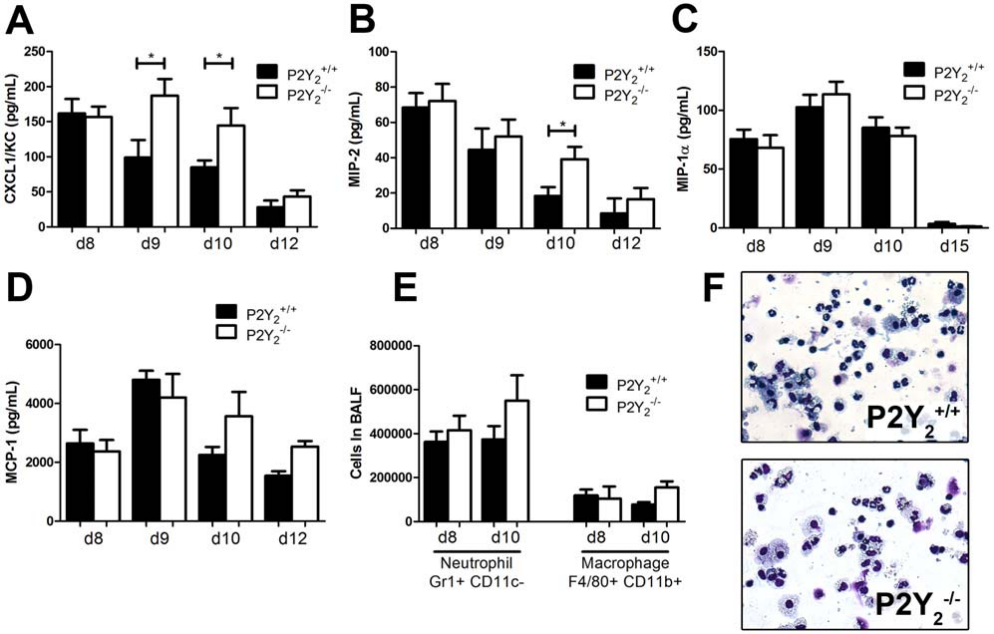
Quantification of neutrophils and macrophages, and their recruiters in the lungs of PVM-infected P2Y_2_^+/+^ and P2Y_2_^−/−^ mice. **A–D.** The level of the chemokines KC/CXCL-1 (A), MIP-2/CXCL-2 (B), MIP-1α/CCL3 (C) and MCP-1/CCL2 (D) was determined by ELISA in the BALFs of PVM-infected P2Y_2_^+/+^ and P2Y_2_^−/−^ mice. (N = 9) **E.** Flow cytometry quantification of neutrophils and macrophages in the BALFs of PVM-infected P2Y_2_^+/+^ and P2Y_2_^−/−^ mice at day 8 and 10 post-inoculation (N = 12). **F.** Cytospin preparations were made from BALFs of P2Y_2_^+/+^ and P2Y_2_^−/−^ mice at day 8 post-infection using a Shandon III cytocentrifuge and were stained using Diff-Quick staining. Magnification: ×400.

We then investigated more precisely neutrophil and macrophage recruitment to the lung in P2Y_2_^+/+^ and P2Y_2_^−/−^ infected mice (Fig. 3). As neutrophils are massively recruited during PVM infection, chemokine KC (CXCL-1) and MIP-2 (CXCL-2) - two major chemokines involved in neutrophils recruitment in mouse - were measured in the BALF of P2Y_2_^+/+^ and P2Y_2_^−/−^ mice by ELISA at days 8, 9, 10 and 12 post-infection. KC levels increased to a peak value at day 9 post-infection in P2Y_2_^−/−^ mice whereas in P2Y_2_^+/+^ mice the level decreased and was significantly lower at day 9 and day 10 (144.6±24.84 N = 13 vs. 84.91±9.918 N = 19 pg/mL, Fig. 3A). MIP-2 levels decreased more rapidly in the P2Y_2_^+/+^ mice than in the P2Y_2_^−/−^ mice and we observed a significant difference between P2Y_2_^+/+^ and P2Y_2_^−/−^ mice at day 10 (respectively, 18.35±5.00 N = 11 vs. 39.14±7.00 N = 9 pg/mL, Fig. 3B). The level of other chemokines such as MIP-1α (CCL3) and MCP-1 (CCL2), respectively involved in the recruitment of neutrophils or monocytes, was determined as well (Fig. 3C and 3D) but their concentration was similar in P2Y_2_^+/+^ and P2Y_2_^−/−^ BALFs.

We then analysed the cellular infiltrates in the lung by flow cytometry analysis of BALF samples obtained at day 8 and day 10 post-infection. The total number of neutrophils and monocytes recovered in BALF was comparable in P2Y_2_^+/+^ and P2Y_2_^−/−^ mice (Fig. 3E). We observed a weak but not significant increase of neutrophils and monocytes in P2Y_2_^−/−^ BALF at day 10 (Fig. 3E). Additionally, cytospin preparations of BALF were performed to identify leukocyte subpopulations at day 8. The neutrophil and macrophage populations observed in the BALFs of P2Y_2_^+/+^ and P2Y_2_^−/−^ mice (Fig. 3F) were counted and these results confirmed the flow cytometry analysis (data not shown).

### Defective infiltration of dendritic cells, and CD4^+^, and CD8^+^ T cells in P2Y_2_^−/−^ inflamed lungs

**Figure 4:**
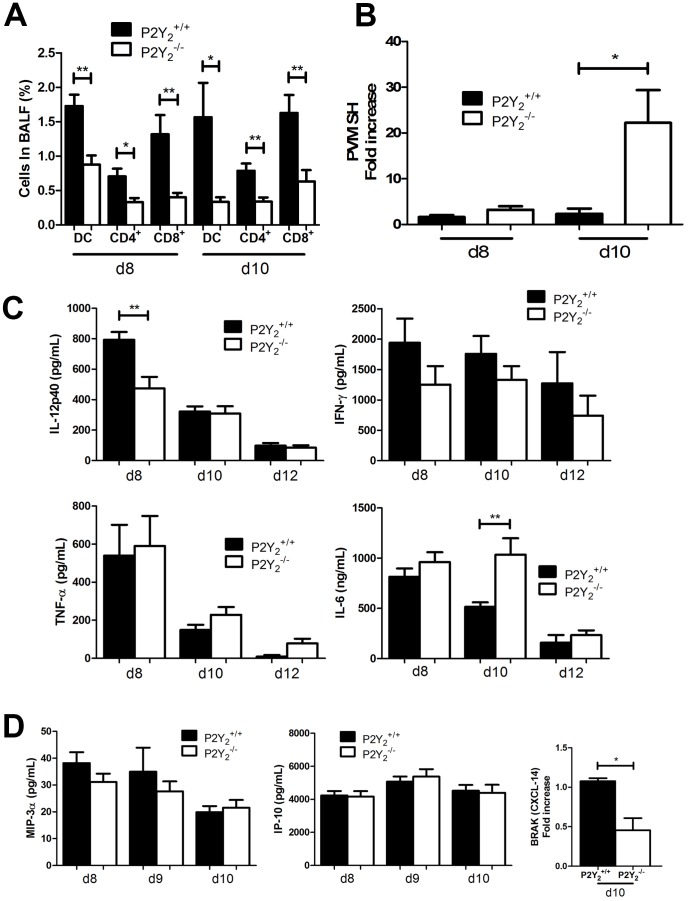
Defective infiltration of DCs, CD4^+^ T cells and CD8^+^ T cells in the lungs of PVM-infected P2Y_2_-deficient mice. **A.** The percentage of DCs (MHC II^+^ CD11c^+^ CD11b^+^), CD4^+^ T cells and CD8^+^ T cells were determined by flow cytometry analysis in BALFs of P2Y_2_^+/+^ and P2Y_2_^−/−^ mice at day 8 and days 10 (d8 and d10) post-infection (N = 5). **B.** Analysis of viral titer in P2Y_2_^+/+^ and P2Y_2_^−/−^ lungs after infection with PVM. PVM viral titer was quantified by quantitative PCR in lung homogenates of P2Y_2_^+/+^ and P2Y_2_^−/−^ mice 8 or 10 days post-infection. Data were normalized to the viral titer obtained for P2Y_2_^+/+^ lungs at d8. **C.** IL-12, IFN-γ, TNF-α and IL-6 levels were determined by ELISA in the BALFs of PVM-infected P2Y_2_^+/+^ and P2Y_2_^−/−^ mice (N = 11). **D.** The level of DC recruiters was determined by ELISA (MIP-3α, IP-10) or qPCR (BRAK) in the BALFs of PVM-infected P2Y_2_^+/+^ and P2Y_2_^−/−^ mice (N = 5).

We have then quantified the number of dendritic cells (DCs), CD4^+^ and CD8^+^ T cells in the BALFs of P2Y_2_^+/+^ and P2Y_2_^−/−^ infected mice. We observed a lower infiltration of these three cell populations in the BALFs of P2Y_2_^−/−^ mice compared to those of P2Y_2_^+/+^ mice at days 8 and 10 after infection (Fig. 4A). PVM viral titer was then quantified by quantitative PCR in lung homogenates of P2Y_2_^+/+^ and P2Y_2_^−/−^ mice at day 8 and day 10 post-infection (Fig. 4B). The data were normalized to the viral titer quantified in P2Y_2_^+/+^ lungs at day 8. Whereas their viral titer was comparable at day 8, a higher PVM viral titer was observed in P2Y_2_^−/−^ lungs compared to P2Y_2_^+/+^ lungs at day 10 (Fig. 4B). We next investigated the production of cytokines involved in the action of immune cells in anti-viral defences. More particularly, IL-12, IFN-γ, TNF-α and IL-6 levels were quantified by ELISA in P2Y_2_^+/+^ and P2Y_2_^−/−^ BALFs at days 8, 10 and 12 post-infection (Fig. 4C). We observed a lower IL-12 level (474.3±75.56 vs. 794.2±50.59 pg/mL) at day 8 and a higher IL-6 level (1034±165.0 vs. 514.8±43.96 ng/mL) at day 10 in the BALFs of P2Y_2_^−/−^ mice compared to those of P2Y_2_^+/+^ mice, respectively (Fig. 4C). The levels of IL-10, IFN-β and IL-17 were also assessed by ELISA but were not detectable in P2Y_2_^+/+^ or P2Y_2_^−/−^ BALFs. We have then realized a comparative microarray analysis of P2Y_2_^+/+^ and P2Y_2_^−/−^ PVM-infected lungs (data not shown). We focused our attention on inflammatory genes and we observed the down-regulation of BRAK (CXCL-14) in P2Y_2_^−/−^ lungs (data not shown). We have thus measured the levels of chemokines implicated in the recruitment of DCs, such as IP-10 (CXCL10), MIP-3α (CCL20) and BRAK (CXCL-14) in P2Y_2_^+/+^ and P2Y_2_^−/−^ BALFs (Fig. 4D). IP-10 and MIP-3α levels were similar in P2Y_2_^+/+^ and P2Y_2_^−/−^ BALFs but we observed a lower BRAK level at day 10 in P2Y_2_^−/−^ BALFs by qPCR (Fig. 4D).

## Discussion

The present study investigated the role of P2Y_2_ receptor in a mouse model of viral pneumonia induced by PVM, the mouse counterpart of human RSV. The two viruses are closely related and provoke similar immune responses. The natural mouse pathogen PVM replicates efficiently following a minimal virus inoculum, and recapitulates many of the clinical and pathologic features of the most severe forms of RSV infection in human infants. By contrast, mice are relatively resistant to infection by human RSV [5]. Besides their phylogenic proximity and genomic similarities, another shared characteristic of the PVM and the RSV pneumoviruses is their capacity to enhance airway hyperreactivity and Th2 response induced after an allergic sensitization and challenge by ovalbumin in mouse [22]. Indeed, PVM infection acts similarly to early-life hRSV infection in human, which is known to increase the risk of subsequent development of childhood asthma.

We demonstrated that P2Y_2_^−/−^ mice display a severe increase in morbidity and mortality rate in response to PVM. The difference in mortality appeared before a difference in body weight was observed between P2Y_2_^+/+^ and P2Y_2_^−/−^ mice, possibly because the rate of weight loss was very high between days 6 and 10 and already maximal in P2Y_2_^+/+^ mice. We investigated first if the lower survival of P2Y_2_^−/−^ mice could be correlated with an excessive inflammation. We observed that several inflammatory chemokines were increased in the BALFs of P2Y_2_^−/−^ mice. More precisely, P2Y_2_^−/−^ mice presented increased levels of KC/CXCL-1 and MIP-2/CXCL-2 chemokines in their BALFs, but no significant higher infiltration of neutrophils or macrophages was observed in the inflamed lungs of P2Y_2_^−/−^ mice until day 10 post-infection.

ATP, released upon tissue damage and concomitant early inflammatory process, constitutes a danger signal, which initiates several pro-inflammatory responses upon binding to purinergic receptors. In particular it has been shown that ATP, released into the airways during asthmatic airway inflammation, can modulate the function of myeloid dendritic cells thereby triggering and maintaining asthmatic airways inflammation [19]. Indeed, DCs are crucial for asthmatic inflammation because they recruit Th2 lymphocytes to the airway wall and trigger local Th2 effector cytokine production. It was also reported that P2Y_2_ receptor exerts a protective role during lung infection such as in the *Pseudomonas aeruginosa* infection model [17].

In the present study, we observed a lower infiltration of DCs, CD4^+^ and CD8^+^ T cells in the BALFs of P2Y_2_^−/−^ mice compared to those of P2Y_2_^+/+^ mice. This lack of infiltration can be correlated to the data of Müller and colleagues demonstrating that P2Y_2_R is involved in the recruitment of DCs in the lungs [23]. IL-12 level was quantified in the BALFs of P2Y_2_^+/+^ and P2Y_2_^−/−^ PVM-infected mice and was significantly lower in P2Y_2_-deficient mice at days 8 and 10 post-infection. DCs are one primary producer of IL-12 which induces the proliferation of NK, T cells, DCs and macrophages, the production of IFN-γ and increased cytotoxic activity of these cells. IL-12 also promotes the polarization of CD4^+^ T cells to the Th1 phenotype involved against viral infection. Higher IL-6 level observed in P2Y_2_^−/−^ BALFs could also reflect a defective Th1 response in these mice. It was indeed shown that IL-6 production by pulmonary dendritic cells impedes Th1 immune responses [24].

The absence of P2Y_2_ receptor and the reduced level of its ligand ATP which are involved in DC recruitment in the lungs [23] could explain lower DC infiltration observed in P2Y_2_^−/−^ lungs. Lower ATP level in P2Y_2_^−/−^ lung could be explained by P2Y_2_-mediated ATP release. P2Y_2_ activation was shown to open pannexin-1 channels forming non-selective pores permeable to ions and large molecules such as ATP in rat carotid body cells [25]. Lower DC and T lymphocyte infiltration could also have been related to reduced level of DC-recruiting chemokines. A comparative gene profiling analysis of P2Y_2_^+/+^ and P2Y_2_^−/−^ PVM-infected lungs focused on inflammatory genes revealed the down-regulation of BRAK (CXCL-14) in P2Y_2_^−/−^ lungs. The quantification of DC recruiters IP-10 (CXCL10), MIP-3α (CCL20) and BRAK (CXCL-14) by ELISA or qPCR in P2Y_2_^+/+^ and P2Y_2_^−/−^ BALFs confirmed lower expression of BRAK at day 10 post-infection in the P2Y_2_^−/−^ BALFs compared to P2Y_2_^+/+^ BALFs. Interestingly, BRAK is a potent chemoattractant and activator of dendritic cells [26]. It has also an ability to block endothelial cell chemotaxis resulting in the inhibition of angiogenesis [27]. CXCL14 is constitutively and highly expressed in many normal tissues, where its source is thought to be fibroblasts [28] and epithelial cells [29] which both express P2Y_2_ receptors. Reduced DC infiltration in P2Y_2_^−/−^ PVM-infected lungs could result from a defect in both direct nucleotide-driven and BRAK-mediated DC chemotaxis. Recruitment of T cells was also affected in PVM-infected P2Y_2_-deficient mice. Both CD4^+^ and CD8^+^ T cells contribute to the clearance of PVM from the lung [11]. Genetically T-cell-deficient or T-cell-depleted mice cannot eliminate PVM.

The increased morbidity and mortality of P2Y_2_^−/−^ mice could be the consequence of a lower viral clearance leading to a more persistent viral load and higher viral titers as observed at day 10 post-infection in the lungs of P2Y_2_^−/−^ infected mice. The decreased viral clearance could result from the defective Th1 response to PVM with a lack of DC and T cell infiltration. Additionally, we cannot exclude that P2Y_2_^−/−^ mice display after 10 days an excessive inflammation with higher neutrophil recruitment compatible with the increase in KC, MIP-2 and IL-6, but this could not be efficiently analysed because of their high and rapid mortality.

In conclusion, our study reveals that the purinergic P2Y_2_ receptor, previously described as a mediator of Th2 response in asthma, is also involved in the initiation of Th1 response protecting mice against lung viral infection.
